# Supplementary material for: Ectocytosis prevents accumulation of ciliary cargo in C. elegans sensory neurons
Source: eLife. 2021 Sep 17;10:e67670. doi: 10.7554/eLife.67670 (PMC8492061; doi:10.7554/eLife.67670)
Supplement: Supplementary file 1. [file elife-67670-supp1.docx]

| Supplementary Table 1. List of strains used in this study | | |  |
| --- | --- | --- | --- |
| Strain | **Background allele** | **Description** | **Source** |
| N2 | N2 | N2 Bristol [ID=WBStrain00000001] | CGC |
| OQ309 | N2 | Ex[pF16F9.3::CFP] | This study |
| OQ276 | N2 | Ex[pGcy-5::TSP-6::wrmScarlet + pF16F9.3::CFP] | This study |
| OQ319 | N2 | Ex[pSra-6::TSP-6::wrmScarlet + pF16F9.3::CFP] | This study |
| OQ280 | N2 | Ex[pGcy-8::TSP-6::wrmScarlet + pF16F9.3::CFP] | This study |
| OQ316 | N2 | Ex[pKlp-6::TSP-6::wrmScarlet + pF16F9.3::CFP] | This study |
| OQ258 | N2 | Ex[pGcy-5::TSP-7::wrmScarlet + pF16F9.3::CFP] | This study |
| OQ235 | N2 | Ex[pOcr-2::XBX-1::mEGFP + pOcr-2::TSP-7::wrmScarlet] | This study |
| OQ205 | N2 | Ex[pKlp-6::TSP-7::wrmScarlet + pKlp-6::mEGFP::let858 3'UTR] | This study |
| OQ157 | N2 | Ex[pKlp-6::TSP-7::wrmScarlet + pUnc-122::GFP] | This study |
| OQ136 | N2 | Ex[pklp-6::mCherry::unc-54 3’UTR] | This study |
| OQ211 | N2 | Ex[pGcy-8::TSP-7::wrmScarlet + pF16F9.3::CFP] | This study |
| OQ200 | N2 | Ex[pGcy-8::GCY-8::wrmScarlet + pF16F9.3::CFP ] | This study |
| OQ266 | N2 | Ex[pGcy-8::SRTX-1::wrmScarlet + pF16F9.3::CFP] | This study |
| OQ272 | N2 | Ex[pSrbc-64::SRBC-64::wrmScarlet + pF16F9.3::CFP] | This study |
| DR86 | *daf-19(m86)* | *daf-19(m86) II.* | CGC |
| OQ334 | *daf-19(m86)* | *daf-19(m86)*; OQ270 | This study |
| OQ359 | *unc-101(m1)* | *unc-101(m1)*; OQ270 | This study |
| GJ3452 | N2 | gcy-22(gj1976[gcy-22::GFP]) V | van der Burght et al., 2020 |
| GJ3461 | *osm-3(p802)* | *osm-3(p802)*; GJ3452 | van der Burght et al., 2020 |
| GOU2366 | *che-3(cas511)* | *che-3(cas511[gfp::che-3(K2935Q)]) I*. | CGC |
| OQ385 | *che-3(cas511)* | *che-3(cas511)*; GJ3452 | This study |
| MX52 | *bbs-8(nx77)* | *bbs-8(nx77) V.* | CGC |
| GJ3457 | *bbs-8(nx77)* | *bbs-8(nx77)*; GJ3452 | van der Burght et al., 2020 |
| OQ387 |  | GJ3452 + Ex[pGcy-5::mKate + pUnc-122::RFP] | This study |
| OQ388 |  | *bbs-8(nx77)*; GJ3452 + Ex[pGcy-5::mKate + pUnc-122::RFP] | This study |
| PLT03 | N2 | [tsp-6::wrmScarlet] CRISPR Knock in | This study |
| OQ366 | N2 | PLT03 + AMsh::CFP | This study |
| OQ368 | *bbs-8(nx77)* | *bbs-8 (nx77)*; PLT03 + AMsh::CFP | This study |
| OQ369 | *osm-3(p802)* | *osm-3(p802)*; PLT03 + AMsh::CFP | This study |
| OQ370 | *che-3(cas511)* | *che-3(cas511)*; PLT03 + AMsh::CFP | This study |
| PR767 | *ttx-1(p767)* | *ttx-1(p767) V.* | CGC |
| OQ171 | *ttx-1(p767)* | Ex[pGcy-8::TSP-7::wrmScarlet + pF16F9.3::CFP] | This study |
| SP1735 | *dyf-7(m537)* | *dyf-7(m537) X.* | CGC |
| OQ252 | *dyf-7(m537)* | Ex[pGcy-8::TSP-7::wrmScarlet + F16F9.3::CFP] | This study |
| OQ231 | N2 | Ex[pGcy-8::TSP-7::wrmScarlet + pGcy-8::mEGFP] | This study |
| OS2248 | N2 | nsIs109 [F16F9.3p::DTA(G53E) + pUnc-122::GFP] | Bacaj et al. , 2008 |
| OQ233 | N2 | OS2248 + Ex[pGcy-8::TSP-7::wrmScarlet + pGcy-8::mEGFP] | This study |
| OQ340 | N2 | OS2248+ Ex[pGcy-8::TSP-7::wrmScarlet + pOsm-3::mEGFP] | This study |
| OQ338 | N2 | Ex[pGcy-8::TSP-7::wrmScarlet + pOsm-3::mEGFP] | This study |
| OQ303 | N2 | Ex[F16F9.3::DYN-1(K46A)::SL2mEGFP] Line 1 | This study |
| OQ304 | N2 | Ex[F16F9.3::DYN-1(K46A)::SL2mEGFP] Line 2 | This study |
| OQ305 | N2 | Ex[F16F9.3::DYN-1(K46A)::SL2mEGFP] Line 3 | This study |
| OQ325 | N2 | Ex[pGcy-5::mKate + pF16F9.3::CFP] | This study |
| OQ327 | N2 | Ex[F16F9.3::DYN-1(K46A)::SL2mEGFP + pGcy-5::mKate] | This study |
| OQ338 | N2 | Ex[pSra-6::mKate + pF16F9.3::CFP] | This study |
| OQ341 | N2 | Ex[pSra-6::mKate + pF16F9.3::DYN-1(K46A)::SL2mEGFP] | This study |
| vyIs46 | N2 | vyIs46[pStr-2::DsRed] | Chiou-Fen Chuang Lab |
| OQ386 | N2 | vyIs46; Ex[F16F9.3::DYN-1(K46A)::SL2mEGFP] | This study |
| OQ329 | N2 | Ex[pGcy-8::mKate + pF16F9.3::CFP] | This study |
| OQ331 | N2 | Ex[F16F9.3::DYN-1(K46A)::SL2mEGFP + pGcy-8::mKate] | This study |
| CB1033 | *che-2(e1033)* | *che-2(e1033) X.* | This study |
| FK135 | *ttx-3(ks5)* | *ttx-3(ks5) X.* | CGC |
| AQ2335 | *lite-1* | *lite-1*; Is[pSra-6Chr2-RFP] | William Schaffer Lab |
| OQ337 | *lite-1* | AQ2335 ; OQ303 | This study |
| OQ270 | N2 | Ex[pGcy-5::GCY-22::wrmScarlet + pF16F9.3::CFP] | This study |
| OQ336 | N2 | OQ303 ; OQ270 | This study |
| MT9958 | *ced-10(n3246)* | *ced-10(n3246) IV.* | CGC |
| OQ312 | *ced-10(n3246)* | *ced-10(n3246)*; OQ270 | This study |
| OQ356 | N2 | Ex[pArrd-4::TSP-6::wrmScarlet + pUnc-122::GFP] | This study |
| VC841 | *alx-1(gk338)* | *alx-1(gk338) III.* | CGC |
| OQ389 | *alx-1(gk338)* | VC841; OQ270 | This study |
|  |  |  |  |

| Supplementary Table 2. List of Molecular Biology / Primers used in this study | | | | | |
| --- | --- | --- | --- | --- | --- |
| Gene | **Fragment type** | **Size (in bp)** | **Description** | **Primer** | **Sequence** |
| pF16F9.3 | Promoter | 588 | Promoter for AMsh, AMso and PHsh glia | Forward | catcaaattcaacaacatgaaatg |
|  |  |  |  | Reverse | attttgtttcttactgtcttgggtatt |
| pGcy-5 | Promoter | 1982 | Promoter for ASER neuron | Forward | tatacatgaaatacatacatagaca |
|  |  |  |  | Reverse | taatttttcgaaaacaataaatagtaaa |
| pGcgy-8 | Promoter | 2186 | Promoter for AFD neuron | Forward | agcaaagggcgtcgattatctcgaa |
|  |  |  |  | Reverse | tttgatgtggaaaaggtagaatcgaaaatc |
| pKlp-6 | Promoter | 1531 | Promoter for 6 pairs of IL2 neurons | Forward | caccaaaaaattcattaaagcatt |
|  |  |  |  | Reverse | cattattctgaaaagttcaactaataa |
| pOcr-2 | Promoter | 2500 | Promoter for AWA, ASH, ADL and ADF neurons | Forward | ttgtacagtttacatttattataggtaggcacta |
|  |  |  |  | Reverse | cttaatgatgtgatgtactctactgataaga |
| pSrbc-64 | Promoter | 1807 | Promoter for ASK neuron | Forward | gttttctaaaaatgagatattactagtg |
|  |  |  |  | Reverse | cagactgtgacaagaaaactgaa |
| pOsm-3 | Promoter | 2511 | Promoter for ASH, ASI and PVQ neurons | Forward | cgaggtttgcttcaaaattcggta |
|  |  |  |  | Reverse | tccgacgcatagctggaaat |
| pArrd-4 | Promoter | 1585 | Pan-ciliary promoter | Forward | ttttcaaatgggcagctcaac |
|  |  |  |  | Reverse | aataggtctcatgaggaaaggg |
| CFP | Fluorescent protein | 870 | Cytoplasmic expression of CFP | Forward | atgagtaaaggagaagaacttttcac |
|  |  |  |  | Reverse | ctatttgtatagttcatccatgcc |
| mEGFP | Fluorescent protein | 872 | Cytoplasmic expression of mEGFP | Forward | atgtccaagggagaggagctct |
|  |  |  |  | Reverse | ctacttgtagagctcgtccattccg |
| mKate | Fluorescent protein | 849 | Cytoplasmic expression of mKate | Forward | atgtccgagctcatcaaggag |
|  |  |  |  | Reverse | ttaacggtgtccgagcttgg |
| mCherry | Fluorescent protein | 864 | Cytoplasmic expression of mKate | Forward | atggtctcaaagggtgaagaagataa |
|  |  |  |  | Reverse | ttacttatacaattcatccatgccacct |
| tsp-6 | Gene | 1577 | From genomic sequence | Forward | atggttcaaggatgtggtaacaaatgc |
|  |  |  |  | Reverse | agcttgggagcgtttctctttg |
| tsp-7 | Gene | 2952 | From genomic sequence | Forward | atggtagaaggaggagttaccatagtta |
|  |  |  |  | Reverse | ataataaaagtcatggaaatccttgaga |
| gcy-22 | Gene | 4621 | From genomic sequence | Forward | atgagtttcatatcaaaatgttttatttgc |
|  |  |  |  | Reverse | gatagattctccattctccttcgc |
| xbx-1 | Gene | 2270 | From genomic sequence | Forward | atgaacatttgggatcttgcca |
|  |  |  |  | Reverse | tcgaacattaatttttgcgattcgat |
| gcy-8 | Gene | 5431 | From genomic sequence | Forward | atgcgaacaaagaaggcattt |
|  |  |  |  | Reverse | tctctgcaatcctgttggatt |
| srtx-1 | Gene | 1299 | From genomic sequence | Forward | atgttggaagatctcttgtacgaagtac |
|  |  |  |  | Reverse | ttcttgatagtagaagctgacagatcg |
| srbc-64 | Gene | 2163 | From genomic sequence | Forward | atgcctgaaatagtaataatcttgaaca |
|  |  |  |  | Reverse | ctgtgaccatgtgagcacag |
| dyn-1 | Gene | 3642 | From genomic sequence | Forward | atgtcgtggcaaaaccaggg |
|  |  |  |  | Reverse | ttatctaggcggtgccatgttg |
| dyn-1(K46A) | Gene | - | K46A substitution mutagenesis | Forward | atcgccgtcgtcggaggacagtccgctggagcgtcgtcggtgc |
|  |  |  |  | Reverse | tgtcctccgacgacggcgatctgtggaagttcgaagctgac |
| wrmScarlet  C-terminal Fusion | Fluorescent protein | 699 | C-terminal fusion wrmScarlet | Forward | atggtcagcaagggagaggcagtta |
|  |  |  |  | Reverse | cttgtagagctcgtccattcctccg |
| mEGFP  C-terminal Fusion | Fluorescent protein | 872 | C-terminal fusion mEGFP | Forward | atgtccaagggagaggagctcttca |
|  |  |  |  | Reverse | ctacttgtagagctcgtccattccg |
| SL2-mEGFP | Fluorescent protein | 1070 | SL2-mEGFP reporter | Forward | ccgctgtctcatcctactttcac |
|  |  |  |  | Reverse | ctacttgtagagctcgtccattccg |
| let-858 3'UTR | Regulatory element | 408 | let-858 3'UTR Regulatory element | Forward | attttcaaattttaaatactgaatatttgt |
|  |  |  |  | Reverse | ccaagcgaggacaattct |

| Supplementary Table 3. List of plasmids used in this study | | |
| --- | --- | --- |
| Plasmid name | **Description** | **Cloning method** |
| AS1 | pF16F9.3::CFP::let-858 3'UTR | Gateway cloning |
| AS2 | pGcy-5::TSP-6::wrmScarlet | Gateway cloning |
| AS3 | pSra-6::TSP-6::wrmScarlet | Gateway cloning |
| AS4 | pGcy-8::TSP-6::wrmScarlet | Gateway cloning |
| AS5 | pKlp-6::TSP-6::wrmScarlet | Gateway cloning |
| AS6 | pKlp-6::mEGFP::let-858 3'UTR | Gateway cloning |
| AS7 | pGcy-5::TSP-7::wrmScarlet | Gateway cloning |
| AS8 | pOcr-2::XBX-1::mEGFP | Gateway cloning |
| AS9 | pOcr-2::TSP-7::wrmScarlet | Gateway cloning |
| AS10 | pKlp-6::TSP-7::wrmScarlet | Gateway cloning |
| AS11 | pF16F9.3::mCherry::unc-54 3'UTR | Gateway cloning |
| AS12 | pGcy-8::TSP-7::wrmScarlet | Gateway cloning |
| AS13 | pGcy-8::GCY-8::wrmScarlet | Gateway cloning |
| AS14 | pGcy-8::SRTX-1::wrmScarlet | Gateway cloning |
| AS15 | pSrbc-64::SRBC-64::wrmScarlet | Gateway cloning |
| AS16 | pGcy-5::GCY-22::wrmScarlet | Gateway cloning |
| AS17 | pGcy-5::GCY-22::mEGFP | Gateway cloning |
| AS18 | pF16F9.3::DYN-1(K46A)::SL2-mEGFP | Gateway cloning |
| AS19 | pGcy-5::mKate::let-858 3'UTR | Gateway cloning |
| AS20 | pGcy-8::mKate::let-858 3'UTR | Gateway cloning |
| AS21 | pGcy-8::mEGFP::let-858 3'UTR | Gateway cloning |
| AS22 | pOsm-3::mEGFP::let-858 3'UTR | Gateway cloning |
| AS23 | pArrd-4::TSP-6::wrmScarlet | Gateway cloning |
