# Supplementary material for: Ectocytosis prevents accumulation of ciliary cargo in C. elegans sensory neurons
Source: eLife. 2021 Sep 17;10:e67670. doi: 10.7554/eLife.67670 (PMC8492061; doi:10.7554/eLife.67670)
Supplement: Transparent reporting form [file elife-67670-transrepform1.docx]

***eLife’s* transparent reporting form**

We encourage authors to provide detailed information *within their submission* to facilitate the interpretation and replication of experiments. Authors can upload supporting documentation to indicate the use of appropriate reporting guidelines for health-related research (see [EQUATOR Network](http://www.equator-network.org/%20)), life science research (see the [BioSharing Information Resource](https://biosharing.org/)), or the [ARRIVE guidelines](http://www.plosbiology.org/article/info:doi/10.1371/journal.pbio.1000412) for reporting work involving animal research. Where applicable, authors should refer to any relevant reporting standards documents in this form.

If you have any questions, please consult our Journal Policies and/or contact us: [editorial@elifesciences.org](mailto:editorial@elifesciences.org).

**Sample-size estimation**

- You should state whether an appropriate sample size was computed when the study was being designed
- You should state the statistical method of sample size computation and any required assumptions
- If no explicit power analysis was used, you should describe how you decided what sample (replicate) size (number) to use

Please outline where this information can be found within the submission (e.g., sections or figure legends), or explain why this information doesn’t apply to your submission:

- No power analysis was used to calculate sample size. Sample sizes varied for each experiment, based on prior experience with similar previous work.

- Sample size can be found within each figure (N). The N indicates the number of animals, number of counted vesicles, number of independent assays, … as indicated in figure legends.

**Replicates**

- You should report how often each experiment was performed
- You should include a definition of biological versus technical replication
- The data obtained should be provided and sufficient information should be provided to indicate the number of independent biological and/or technical replicates
- If you encountered any outliers, you should describe how these were handled
- Criteria for exclusion/inclusion of data should be clearly stated
- High-throughput sequence data should be uploaded before submission, with a private link for reviewers provided (these are available from both GEO and ArrayExpress)

Please outline where this information can be found within the submission (e.g., sections or figure legends), or explain why this information doesn’t apply to your submission:

The confocal images provided are representative of 6 to 10 individuals imaged on same or different days; these individuals originate from 3 independent transgenic lines, equally distributed.

Figure 2 and 3: Data quantification was done pooling the datapoints for 3 biologically independent lines, using the same confocal settings and conditions. For Figure 4, 5, 7: Data quantification was done pooling the datapoints of 3 independent experiments performed on different days, using the same confocal settings and conditions. All data points were taken into account and no outliers were discarded for posterior analysis.

**Statistical reporting**

- Statistical analysis methods should be described and justified
- Raw data should be presented in figures whenever informative to do so (typically when N per group is less than 10)
- For each experiment, you should identify the statistical tests used, exact values of N, definitions of center, methods of multiple test correction, and dispersion and precision measures (e.g., mean, median, SD, SEM, confidence intervals; and, for the major substantive results, a measure of effect size (e.g., Pearson's r, Cohen's d)
- Report exact p-values wherever possible alongside the summary statistics and 95% confidence intervals. These should be reported for all key questions and not only when the p-value is less than 0.05.

Please outline where this information can be found within the submission (e.g., sections or figure legends), or explain why this information doesn’t apply to your submission:

Statistical tests used in this manuscript are described in “Statistical analysis” in Materials and methods section as well as in figure legends. Scatterplots were used to display data distribution, mean and standard deviation are reported in graphs in such plots.

For experiments with high N number (>100) scatterplot was substituted for box and whiskers plot representing min/max values and the median value (Figure 4B, Figure 6_Figure Supplement 1).

p values are displayed in figures, n.s. describes non significance for the specified comparison.

(For large datasets, or papers with a very large number of statistical tests, you may upload a single table file with tests, Ns, etc., with reference to sections in the manuscript.)

**Group allocation**

- Indicate how samples were allocated into experimental groups (in the case of clinical studies, please specify allocation to treatment method); if randomization was used, please also state if restricted randomization was applied
- Indicate if masking was used during group allocation, data collection and/or data analysis

Please outline where this information can be found within the submission (e.g., sections or figure legends), or explain why this information doesn’t apply to your submission:

Animals were grouped based on genotype and/or condition (developmental stage, treatment, etc) as indicated in figure and figure legends. Supplementary Table 1 lists the different genotypes used in this study.

**Additional data files (“source data”)**

- We encourage you to upload relevant additional data files, such as numerical data that are represented as a graph in a figure, or as a summary table
- Where provided, these should be in the most useful format, and they can be uploaded as “Source data” files linked to a main figure or table
- Include model definition files including the full list of parameters used
- Include code used for data analysis (e.g., R, MatLab)
- Avoid stating that data files are “available upon request”

Please indicate the figures or tables for which source data files have been provided:

Figure 1, Figure 2, Figure 5, Figure 6, Figure 7, Figure 8, include Figure Supplements that have been attached to the manuscript.
